# Effect of recreational sport and physical activity participation on well-being during early parenthood: a randomized controlled trial

**DOI:** 10.1093/abm/kaae081

**Published:** 2024-12-09

**Authors:** Ryan E Rhodes, Mark R Beauchamp, Valerie Carson, Sandy Courtnall, Colin M Wierts, Chris M Blanchard

**Affiliations:** University of Victoria, Victoria, British Columbia, V8P 5C2, Canada; University of British Columbia, Vancouver, British Columbia, V6T 1Z4, Canada; University of Alberta, Edmonton, Alberta, T6G 2R3, Canada; University of Victoria, Victoria, British Columbia, V8P 5C2, Canada; University of Victoria, Victoria, British Columbia, V8P 5C2, Canada; Dalhousie University, Halifax, Nova Scotia, B3H 4R2, Canada

**Keywords:** parenthood, well-being, mental health, psychosocial health, family function, relationship satisfaction

## Abstract

**Background:**

Parents with children in the home may benefit considerably from sport participation, given the high levels of physical inactivity and psychosocial distress among this group. The purpose of this study was to evaluate the effectiveness of team sport participation on mental health (primary outcome) as well as other secondary psychosocial outcomes compared to an individual physical activity condition and a “date night” control condition among parents with young children (under the age of 13).

**Methods:**

A three-arm parallel design single blinded randomized controlled trial compared the team sport (*n* = 58), individual physical activity (*n* = 60), and control condition (*n* = 66) over three months. Well-being variables (short-form-12, satisfaction with life scale, parental stress scale, relationship assessment scale, family inventory version II) were assessed at baseline and post-randomization at 6 weeks and 3 months. Rolling recruitment began in winter 2016 until spring 2023. Analyses were conducted using generalized linear mixed models.

**Results:**

Team sport participation resulted in improvements in mental health and increased relationship satisfaction compared to the other conditions. Team sport participation also showed improvements in lowering parental stress and increasing family emotional expressiveness compared to the control condition. All conditions improved satisfaction with life, lowered stress, increased relationship satisfaction, benefited family health/competence and lowered family conflict over time.

**Discussion:**

The findings extend prior observational research by demonstrating team sport participation may be a viable activity to recommend for parents of young children, who are typically challenged by lower well-being, stress, and social isolation from other adults.

**Registered trial:**

The clinical trial is registered with the National Library of Medicine at the National Institutes of Health registration ID is NCT02898285.

## Teaser

Parents with young children who participated in recreational team sport had improvements in mental health and increased relationship satisfaction compared to parents who participated in individual physical activities or a “date night” control condition.

## Background

Regular moderate-to-vigorous intensity physical activity among adults is associated with reduced complications of over 25 chronic health and wellness conditions,^1^ and improved social outcomes such as lowered loneliness and greater community connectedness.^2,3^ Recreational sport participation may be a particularly viable form of regular physical activity for numerous health and well-being benefits. Specifically, recreational sport participation among adults is associated with several important psychosocial outcomes, including psychological well-being/life satisfaction, lower stress, higher social functioning, greater vitality, enjoyment, and a sense of community belonging when compared to adults who do not participate in sport.^2,4,5^ In addition, team sport participation is associated with even higher levels of these outcomes when compared to adults who participate in individual sport.^6^ It has been suggested that the social nature of team sport may account for these added benefits.^2^ The only study that has compared sport to other general forms of physical activity found that sport participation was associated with higher psychosocial outcomes—particularly lowered stress and distress—compared to any other type of occupational or leisure physical activity.^7^ Similar to the findings about team sport, the study also identified that the main reason for the higher psychosocial outcomes was the enjoyment and social aspects of sport and these outcomes were associated with as little as 20 min of sport participation per week.^7^ Clearly, this is an impressive and consistent series of observational findings that demonstrate a potential link of team sport to positive psychosocial outcomes among adults. Sport participation may also serve the dual aim of increasing physical activity to reap the well-established physical *and* psychological benefits associated with this behavior, such as reduced risk for chronic disease, reduced depression and anxiety, and improved overall quality of life.^8^

Unfortunately, despite this positive early-phase research on the multitude of benefits from sport participation, many adults do not participate in sport. For example, even among the regions of the world where sport participation is most prevalent, such as Australia (53% participation;^9^), Scandinavia (42% participation)^10^ and countries in northern Europe (40%),^10^ many adults report that they do not engage in recreational sport.^11^ In Canada, the context in which the current trial was undertaken, sport participation declines to 23% in adulthood from over half of Canadians participating in sport during late adolescence (55% participation).^12^

When considering the suboptimal participation rates and the potential psychosocial benefits of adult sport together, it should be noted that some adult demographic groups may accrue a larger benefit than others. One of these groups is parents with children residing in the family home. Dramatic effects of increased depression, anxiety, stress, and feelings of parenting unworthiness are often reported in this phase of parenting, with relatively equal effect on mothers and fathers.^13,14^ Post-partum depression, for example, ranges from 8% to 28% for mothers and fathers respectively.^15,16^ While these negative psychosocial consequences tend to be heightened between 3 and 6 months post-partum,^13,17^ it is noteworthy that elevated levels of depression appear to persist among parents several years into parenthood when compared to age-matched non-parents.^18^ Similarly, negative psychosocial functioning can persist during the child rearing years, that comes with the addition of increased loneliness and social isolation (from other adults) and is compounded by occupational stress and financial costs.^19–22^ Most studies also demonstrate that fatigue and parenthood are related.^23^ The finding is associated with sleep disruption^24,25^ and it appears to hold for both mothers and fathers.^23^ Fatigued parents are also at risk for mental and physical health problems that extend to affecting their employment, feelings of parental competence, and marital/social relationships.^22,23^ Thus, interventions that can promote psychosocial well-being among parents are warranted. Given the associations between sport participation and improved well-being, reduced distress and increased social functioning and vitality,^2,4,5^ parents may be an ideal group to promote sport participation. At present, no research has examined whether sport participation can improve these psychosocial outcomes that are often negatively affected by the parenting experience.

Parents report significant declines in overall physical activity and this includes sport participation,^26–28^ making this population an important focus for sport and physical activity promotion. The demands of parenthood likely influence lifestyle changes, which in turn may compromise the pursuit of various physical activities.^26,29^ Specifically, sport participation decreases by as much as 50% during the child-rearing years across most countries,^10,30,31^ and statistics show that participation in sport rarely resumes after these years.^30,32^ Clearly, parents represent an important sport promotion demographic that may stand to gain considerable psychosocial benefits from participation.

To date, there has been no experimental investigation into the effects of team sport participation on key psychosocial outcomes in any adult population. Indeed, systematic reviews on adult sport participation and psychosocial outcomes concluded that experimental research is desperately needed to substantiate a cause-effect relationship.^2,4,5,33^ Furthermore, the effect of individual physical activity versus team sport has not been investigated empirically with parents. This is a noteworthy limitation of prior research, given the promising effects of sport participation on psychological and social health. Therefore, the purpose of this study was to evaluate the effectiveness of team sport participation on mental health (primary outcome) as well as other key potential psychosocial secondary outcomes (parenting stress, satisfaction with life, relationship satisfaction, family functioning) compared to an individual physical activity condition and a “personal time/date night” control condition (a suitable comparator as it is time spent away from children that is not active in nature) among parents with children residing in the home.^34^

Based on the current observational evidence^2,4,5,33^ we hypothesized that participants randomized to the team sport condition will display better mental health (primary trial outcome) and psychosocial functioning (secondary outcomes) when compared to the two other conditions after 3-months of participation (primary end-point). Furthermore, we hypothesized that participants in the individual physical activity condition will display better mental health and psychosocial functioning compared to those randomized to the control condition after 3-months of participation.

## Methods

The full detailed methods for this study are reported elsewhere.^34^ The study was approved by the University of Victoria Human Research Ethics Board and all participants provided informed consent prior to being enrolled. The design, conduct, and reporting of the trial followed the Consolidated Standards of Reporting Trials guidelines^35^ and the Standard Protocol Items: Recommendations for Interventional Trials guidelines.^36^ The trial was registered with the Clinical Trials Registry at the National Library of Medicine Identifier: NCT02898285.

### Design

This study utilized a three-arm parallel design, single blinded randomized controlled trial design. Specifically, participants were assigned to one of three groups: (1) team sport condition; (2) individual physical activity condition; and (3) “date night” control condition for a duration of 3-months (primary end-point). Outcome measures in all three groups were assessed at baseline, and post-randomization at 6-weeks, and 3-months. Randomization was performed by the project coordinator using Excel Sheet Randomization at a 1:1:1 allocation ratio. A research assistant then created prepackage envelopes containing certificates that indicated the condition assignment. A second research assistant, who was blind to the participant’s condition, presented the participants with their randomized allocation. Parent couples who wished to participate in the study were randomly assigned at the level of the couple to reduce experimental contamination. Recruitment began in November 2016 set up in three 3-month-waves throughout the year (fall, winter, and spring) due to the start of the team sport season. Recruitment was officially put on hold effective April 2020 due to the COVID-19 pandemic. We were able to resume recruitment in April 2022 until our end date of April 2023.

### Participants

#### Inclusion criteria

Parents were considered eligible if they reported at least one child under 13 years of age who resides in their home. The decision for this criterion was based on the considerable care needs of children and the subsequent time constrains during this time that challenge parental pursuit of physical activity.^13,17,25,37^ Both mothers and fathers were eligible, inclusive of those with single-parent status. Parents, however, needed to report that they were not engaging in regular sport participation (operationally defined as having not participated in an organized sport in the month prior to baseline) and being below the recommended physical activity guidelines for public health (150 minutes of weekly moderate to vigorous intensity physical activity);^38^ at baseline, through simple assent (yes/no) at the study screening. Parents also needed to be willing to participate in a team sport in addition to being deemed safe to engage in moderate intensity physical activity as assessed via the Get Active Questionnaire.^39^

Participants were recruited primarily through advertisements via online interest sites and social media platforms. Specifically, posts targeted for a local parent audience were advertised weekly on Facebook and Instagram. Additionally, print advertisements were placed at recreation centers, health care centers, children’s recreation classes, and coffee shops monthly. Pamphlets were also offered at bi-weekly booths at local community markets and family-oriented events. To ensure diversity of the study population, greater Victoria was systematically stratified into regions. Facilities from each region were randomly selected and contacted for recruitment. In addition to these recruitment tactics, we also engaged in a referral system, whereby current participants were invited to pass on study information to others. Finally, incentives for participation included honorariums subsidizing the cost of the activity (up to a total of $80 CDN) as well as assistance with childcare costs (up to $25 CDN weekly).

#### Study setting

Participants were recruited in the Capital Region District, British Columbia, Canada.

### Interventions

Participants randomized to the *team sport condition* were asked to make a selection from a customized and up-to-date handout of our a priori environmental scan of the available adult team sport programs in greater Victoria, Canada. These participants were instructed that their team sport choice should follow the basic definition of team sport by Eime and colleagues (2010), where at least two participants are active in a necessary collaboration to achieve the objective of the physical activity within a set of rules. To be inclusive of autonomy of choice to as many nontraditional activities as possible, we indicated that the activity could forego traditional team sport (eg, basketball, soccer) but it needed to have a set temporal structure (scheduled time/day each week), leadership structure (include a coach or instructor), and have the same group involved each week (social structure). These types of activities included, but were not limited to basketball, soccer, volleyball, dragon boating, kickboxing classes, running groups, bootcamps, and so forth.

Participants randomized to the *individual physical activity condition* were provided with a customized handout of our environmental scan of possible adult individual physical activities and asked to choose from one of these available options. Individual physical activity was defined as a physical activity performed or completed alone with a structured progression toward an outcome or goal. Participants in this group were instructed that their activity must be done on their own (ie, if they choose running, they cannot sign up with a running group). Such individual physical activities included rowing, running, resistance training, cycling, swimming, rock climbing, etc. In some cases (see Supplementary Table S1), a participant specifically asked if they could do a yoga class for their individual physical activity and were advised this was acceptable so long as they minimized interactions and socializing with other participants.

To retain some control of the sport intensity, the sport and physical activities for both of these conditions had to fall within the moderate intensity range for metabolic equivalent ratings (3.5-6.0).^40^ To improve adherence to these programs, we also engaged the participants of both these conditions in a planning process. The material for planning was based on several streams of prior work in the adult physical activity literature and our prior trials.^41,42^ Participants were instructed to plan for “when,” “where,” “how,” and “what” sport participation will be performed commensurate with the creation of action planning^43,44^ as well as to elicit and then strategize around barriers commensurate with coping planning.^45,46^ Participants in the team sport and individual physical activity conditions were asked to keep track of their attendance, aligning with self-monitoring.^47^

Finally, participants in the “date night” *control condition* were asked to use the honoraria funds to treat themselves to a weekly “night out” or “personal time” of their choice (ie, with a friend, with a partner, or by themselves), so long as the time is not physically active and the time is spent away from their children. Examples for personal time were provided, such as going for coffee, going to the movies, board game cafés, taking an arts and crafts workshop or cooking class. Participants in the “date night” control condition were told they will not receive any more contact until the next assessment period. All participants, for all conditions, were asked to commit to their assigned activity at least one night (or day) per week for at least one hour for the 3-months of the study.

### Outcome measures

Assessments of all outcomes were collected through online questionnaires (available on SurveyMonkey).

### Primary outcome

*Mental health* was assessed using the six item sub-scale from the short-form 12 (SF-12) health survey^48^ which measures a composite of mental health-related quality of life on a range of functional domains including vitality, social functioning, emotional well-being, depressive symptoms, and anxiety symptoms.^49^ A higher score is indicative of higher vitality, social functioning, and emotional well-being, and lower depressive symptoms, and anxiety symptoms. The SF-12 has been used extensively with adult populations^48,49^ and in this study the scores derived from this measure exhibited acceptable internal consistency across baseline (α = .67), 6-week (α = .80), and 3-month assessments (α = .79).

### Secondary outcomes

*Overall life satisfaction* was measured with the satisfaction with life scale.^50^ This five-item measure has been used in prior adult sport research,^51^ and was scored on a seven-point Likert-type scale, from 1 (strongly disagree) to 7 (strongly agree). A higher score is indicative of higher life satisfaction. Example items for the measure include “the conditions of my life are excellent” and “so far I have gotten the important things I want in life.” The measure showed acceptable internal consistency scores across baseline (α = .88), 6-week (α = .92), and 3-month assessments (α = .90).

Given our parent sample, *parental stress* was measured with the 18 item parental stress scale,^52^ scored from 1 (strongly disagree) to 5 (strongly agree) on a Likert scale. Example items include “It is difficult to balance different responsibilities in my life because of my child(ren),” “If I had to do it over again, I might decide not to have children,” and “I feel overwhelmed by the responsibility of being a parent.” A higher score is indicative of higher perceived stress. The measure showed acceptable internal consistency scores across baseline (α = .86), 6-week (α = .85), and 3-month assessments (α = .87).

Parents in our sample who reported being in a current relationship with a romantic partner also completed the *Relationship Assessmen*t Scale,^53,54^ which is a seven item generic measure of relationship satisfaction, scored on a seven point scale from 1 (low satisfaction) to 7 (high satisfaction). A higher score is indicative of better relationship satisfaction. Example items include “how well does your partner meet your needs,” and “in general, how satisfied are you with your relationship?.” The measure showed acceptable internal consistency scores across baseline (α = .92), 6-week (α = .95), and 3-month assessments (α = .94).

Finally, family functioning was assessed using four subscales of the 36 item self-report Family Inventory Version II^55,56^: familial health/competence (eg, “we all have a say in family plans”), low conflict (eg, “there is a closeness in my family but each person is allowed to be special and different”), cohesion (eg, “our happiest times are at home”), and emotional expressiveness (eg, “family members pay attention to each other’s feelings”). A higher score is indicative of higher values in familial competence, cohesion, and emotional expressiveness, and lower conflict. The measure is completed using a five-point scale ranging from 1 (does not fit our family) to 5 (fits our family well). The measure showed acceptable internal consistency scores across baseline (competence α = .92; cohesion α = .70; conflict α = .88; expressiveness α = .81), 6-week (competence α = .90; cohesion α = .78; conflict α = .87; expressiveness α = .81), and 3-month assessments (competence α = .89; cohesion α = .72; conflict α = .85; expressiveness α = .81).

### Demographics and health behaviors

A brief section in the baseline questionnaire was used to assess demographic characteristics including age, gender, marital status, ethnicity, level of education, health background, employment information, sleep, smoking, and physical activity behavior, measured using the Godin Leisure-Time Exercise Questionnaire,^57^ adapted with an open scaling of duration so that total minutes of moderate-vigorous intensity physical activity could be calculated.^58^ Child-related demographic information was also collected, including the number of children in the home.

### End of trial fidelity evaluation

The brief trial process evaluation of intervention fidelity at the 3-month assessment included a question about adherence of participants to their respective condition, four questions that asked participants to rate the satisfaction of their intervention, and the participant’s intention to conitnue with the activities. Adherence had five options, ranging from (1) did not adhere at all, to (5) did not miss a session. Responses for the satisfaction questions were provided on a 5-point Likert scale with anchors ranging from (1) strongly disagree to (5) strongly agree. The questions designed to assess intervention satisfaction included those related to participants’ perceived enjoyment, perceived benefit, positive anticipation each week, and perceived challenges associated with participation. Finally, participants intentions to continue with the activity (eg, team sport, individual activity, date nights) after the trial ends was assessed using a decisional intention^59^ format with a yes/no dichotomy.

### Procedures

Those interested in taking part in the study were invited to contact the project coordinator via email or phone, as noted on the recruitment information. At the next stage of the enrollment process, potential participants were formally screened over the phone by the project coordinator.

After interested participants were deemed eligible and enrolled, they were booked for a baseline meeting. At this meeting, informed consent was obtained by a research assistant, and participants were asked to complete the baseline questionnaire. Next, participants were randomized into one of the three conditions. Specifically, a research assistant, who was blind to each participant’s condition, presented the participant with a prepackaged envelope, which revealed the condition allocation. The research assistant then outlined the possible activity options and provided resources to facilitate registration into an activity program (when the participant was allocated to the team sport and individual physical activity conditions). Because enrollment took place in waves that align with seasons for adult sport leagues/programs, those who demonstrated interest were placed on a waitlist to enroll during the uptake for the next wave. After the initial six weeks of intervention, participants were sent follow-up questionnaire links to complete via email. Contact was made initially with a phone call by a research assistant to answer any questions the participants had about their experiences over the last 6-weeks. A similar protocol was followed at the 3-month trial end point.

### Analyses

All analyses were conducted using SPSS (28.0). Little’s missing completely at random (MCAR) test^60^ was conducted to examine the pattern of missing data over time; this included baseline demographics and all outcome measures at baseline, 6-weeks and 3-months. Then, to determine whether the psychosocial outcomes changed over time similarly for all three conditions, a series of generalized linear mixed models (GLMMs) were conducted. A strength of this approach is that participants are included in the analysis if they provide at least one data point along the trajectory.^61,62^ Given the hierarchical nature of the data (ie, the 3 measurement occasions at Level-1 were considered to be nested within the participant at Level-2), the Optimal Design (OpDes) program for power estimation of hierarchical linear models^61^ was used. With an alpha of 0.05, three measurement occasions, three groups, and a small-medium effect size (.40), a total of 207 participants (ie, 67 parents per condition) were needed to show significant differences over time [See also^34]^.

The first GLMM included a random intercept, condition treated as a categorical variable (0 = Date night control; 1 = Individual physical activity; 2 = Team Sport), a continuous linear trend (centered at baseline), a condition × linear trend interaction (date night was the comparison group at baseline) and dyad (0 = partner not in study; 1 = partner in study) to control for having a partner (or not) in the study. We also considered whether to include recruitment year (pre-COVID 19 v. post-COVID 19) as a covariate. Of the 204 participants that were recruited, 28 were recruited from 2022 to 2023, whereas 176 were recruited from 2016 to 2019. When examining the distributions by condition, the chi-square was nonsignificant χ^2^ (2) = .22, *P* = .90. However, the cell sizes were quite small for participants that were recruited from 2022 to 2023 across conditions [“Date night” = 11, Individual sport = 9, Team sport = 8]. Therefore, we did not include year as a covariate in the main linear mixed models. A pairwise comparison was also included to compare team sport vs. individual physical activity for a given outcome. In this model, date night was compared to individual physical activity and team sport at baseline for a given outcome and the interaction indicated whether the change in a given outcome over time is similar by condition. The next model included a random linear trend and Akaike’s Information Criteria (AIC^63^) was used for model comparison (with smaller values representing a better fit). Once the final model was identified, Pearson residuals were examined^64^ and variables were transformed if needed (eg, if the residuals were not normally distributed) to improve the model fit by squaring the distribution or via winsorization (ie, by replacing outliers identified using boxplots and z-scores >3.29 with the next highest value in a distribution for a given condition at a given time point).^65^ Next, significant linear trends were followed up to examine changes from baseline to 6-weeks, baseline to 3-months, and 6-weeks to 3-months. Subsequent models then centered the linear trend at 6-weeks or 3-months to examine condition effects at each time point, aligned with our a priori analysis plan.^34^ Effect size *d*^66^ estimates were calculated to complement the statistical analyses. Finally, end of trial evaluations were explored using basic descriptives, followed by one-way analyses of variance and Chi-squared (χ^2^) analyses by condition.

## Results

### Participant flow

**Figure 1.**
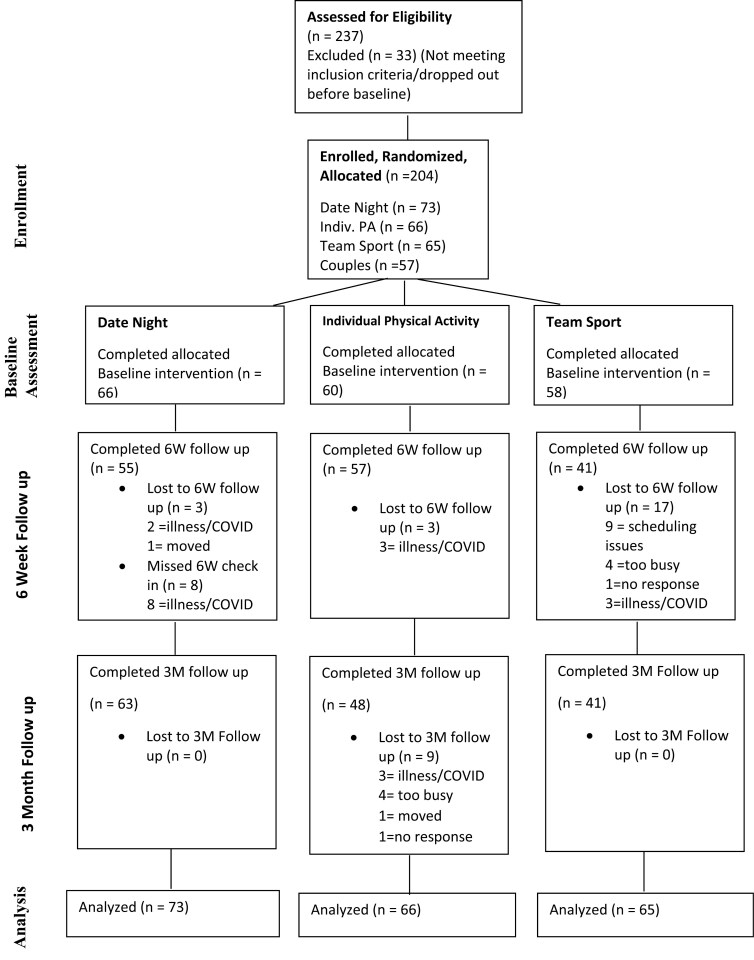
Study flow from recruitment to analyses.

Two hundred and thirty-seven participants contacted the research team about participating in the study. Of these, 33 were subsequently uninterested, could not be reached, or were not eligible. The 204 participants who met the inclusion criteria completed baseline assessments and were randomly assigned to one of the three conditions (Figure 1; *n* = 65 in the team sport condition; *n* = 66 in the individual physical activity condition, and *n* = 73 in the control condition). Furthermore, the specific sport and physical activities chosen by participants in these groups, respectively, can be found in Supplementary Table S1. Of the 204 participants at baseline, 41 participants in the team sport condition, 48 participants in the individual physical activity condition, and 63 in the control condition completed the study to the 3-month end-point (75% retention). The distribution of couples by condition was nonsignificant χ^2^ (2) = 1.39, *P* = .50. Of note, due to an internet software migration error at our University systems management, we were unable to retrieve the baseline data of 20 participants. There was no significant difference among group assignment in this missing data (*P* > .25). Other than this system migration failure, the reasons listed for dropping out of the study included difficulties with scheduling team sport activities (*n* = 9), too busy (*n* = 8), illness/COVID (*n* = 11), moving cities (*n* = 2), and unable to contact (*n* = 2). No participants cited harms associated with the study.

### Baseline characteristics of respondents

Participants reported a mean age of 37.73 years (SD = 7.12), with 67.6% female representation. Most participants were white (83.6%), university educated (75%), married or common law (89.1%), employed (66.3%), and above the median income for Canadian adults (see Table 1). On average, parents reported minimal health conditions. For health behaviors, participants reported an average of 6.82 (SD = 1.07) hours of sleep, were nonsmokers (96.2%), and mean MVPA was 140.65 (SD = 147) min.

**Table 1.** Baseline demographic, health, and behavior profile.

Characteristic	Control condition (*n* = 73)	Individual PA condition (*n* = 66)	Team sport condition (*n* = 65)
Demographic profile			
Age mean (*SD*)	37.37 (7.26)	38.16 (6.27)	37.70 (7.90)
% female	69.2	67.8	65.5
% visible minority (excluding aboriginal)	15.3	13.3	20.7
% aboriginal	2.0	3.0	0.0
% completed university	71.2	73.3	81.0
% >$75,000 CAN family income	66.1	75.6	59.6
% currently employed	68.2	62.5	61.8
% married/common law	80.8	91.3	86.2
% who had a partner in the study	54.8	51.5	55.9
Mean # of children in the home (SD)	1.57 (0.71)	1.90 (0.82)	1.57 (0.57)
Health profile			
Mean hours of sleep/Night (*SD*)	6.73 (0.98)	6.92 (1.14)	6.83 (1.11)
Mean MVPA min self-report (*SD*)	160.89 (166.64)	129.75 (130.33)	128.91 (139.67)
% smoker	4.5	6.7	0.0
% with heart disease	0.0	0.0	0.0
% with diabetes	2.7	5.2	1.8
% with cancer	4.5	5.2	3.5
% with high blood pressure	12.3	3.4	3.6
% with high cholesterol	4.1	8.6	3.6

Abbreviations: *SD*, standard deviation; PA, physical activity.

### Data preparation

Little’s test suggested the data was MCAR [χ^2^ (355) = 353.71, *P* = .51] and supported the use of GLMMs. Next, examination of model residuals from the GLMMs resulted in the distributions for satisfaction with life and relationship satisfaction being squared and distributions for competence and expression being winsorized to improve the models (see Supplementary Table S2 for the marginal means for the transformed and nontransformed variables and Supplementary Table S3 for all effect sizes of the analyses).

### Main outcome: mental health

For the primary outcome of *mental health*, the final model (AIC = 3692.52) included a random intercept and slope (intercept variance = 160.34, *P* < .01; linear trend variance = 38.89, *P* < .01; covariance = −66.03, *P* < .01) and showed the linear trend was nonsignificant (Table 2). However, follow-up analyses showed that the team sport condition had significantly higher mental health compared to the date night condition at the primary end-point of 3-months (beta = 5.38, *P = *.01; *d* = .49), and at the interim assessment at 6-weeks (beta = 4.68, *P* = 01; *d* = .48). The team sport condition was also higher at our primary end-point of 3-months compared to the individual physical activity condition (beta = 3.69, *P* < .047; *d* = .35).

**Table 2.** Results from the generalized linear mixed models for mental health.

	Centered at baseline		Centered at 6-weeks		Centered at 3-months	
Parameter	Beta^b^	95% CI	Beta^b^	95% CI	Beta^b^	95% CI
Intercept	41.72^a^	37.69, 45.76	41.32^a^	38.52, 44.12	40.92^a^	38.17, 43.67
Condition						
Date night	0		0		0	
Individual PA	2.02	−2.80, 6.85	1.86	−1.33, 5.05	1.69	−1.80, 5.19
Team sport^c^^,^^d^^,^^e^	3.98	−1.04, 8.99	4.68^a^	1.43, 7.93	5.38^a^	1.67, 9.10
Linear trend	−.40	−2.42, 1.62	−.40	−2.42, 1.62	.40	−1.62, 2.42
Condition × linear trend						
Date night × linear trend	0		0		0	
Individual PA × linear trend	−.17	−2.93, 2.59	−.17	−2.93, 2.59	.17	−2.59, 2.93
Team sport × linear trend	.70	−2.27, 3.68	.70	−2.27, 3.68	−.70	−3.68, 2.27
Dyad						
Do not have a partner in study	0		0		0	
Have a partner in study	1.68	−.84, 4.21	1.68	−.84, 4.21	1.68	−.84, 4.21

Abbreviations: CI, confidence interval; PA, physical activity.

^a^ *P* < .01.

^b^Betas are based on the original distribution.

**Pairwise Comparisons by Condition**.

^c^At baseline, team sport = individual PA (contrast estimate = 1.95, 95% CI [−2.77, 6.68]).

^d^At 6-weeks, team sport = individual PA (contrast estimate = 2.82, 95% CI [−.24, 5.88]).

^e^At 3-months, team sport > individual PA (contrast estimate = 3.69*, 95% CI [.05, 7.32]).

### Secondary outcomes

*Overall satisfaction with life* (AIC = 3497.27; intercept variance = 96.45, *P* = .01) showed a significant linear trend (Supplementary Table S4). Follow-up analyses showed that baseline scores were significantly lower (across conditions) compared to 6 weeks (beta = 3.65, *P* = .02) and 3-months (beta = 2.24, *P* = .00); however, there was no difference between 6-weeks and 3-months (beta = 1.37, *P* = .20). There were no condition effects.

The final model for *Perceived stress* (AIC = 364.98; intercept variance = .19, *P* < .01; linear trend variance = .01, *P* = .02; covariance = −.12, *P* = .01) showed that the linear trend was significant (Supplementary Table S5). Follow-up analyses showed that baseline scores were similar to 6-weeks (beta = −.06, *P* = .20), yet higher compared to 3-months (beta = −.08, *P* < .01). Perceived stress was also significantly higher at 6-weeks compared to 3-months (beta = −.13, *P* = < .01). Further, participants randomized to the date night control condition had significantly higher scores compared to team sport at 6-weeks (beta = −.16, *P* = .04; *d* = .36) and higher scores at the primary end-point of 3-months (beta = −.17, *P* = .045; *d* = .32).

For *Relationship satisfaction*, the final model (AIC = 3196.77; intercept variance = 95.11, *P* < .01) showed a significant linear trend (Supplementary Table S6). Follow-up analyses showed that (across conditions) 3-month end-point scores were significantly higher compared to baseline (beta = 1.44, *P* = .02) and 6-weeks (beta = 2.78, *P* = .03), whereas baseline scores were similar to 6-weeks (beta = .20, *P* = .84). Further, team sport had significantly higher scores compared to date night at baseline (beta = 5.31, *P* = .01; *d* = .44), and at 6-weeks (beta = 4.78, *P* = .01; *d* = .44) and 3-months (beta = 4.25, *P* = .04; *d* = .35). Team sport also had significantly higher scores compared to the individual sport condition at baseline (beta = 4.03, *P* = .04; *d* = .36), and at 6-weeks (beta = 4.19, *P* = .02; *d* = .41) and 3-months (beta = 4.34, *P* = .04; *d* = .36).

Finally, *family function* sub-scores of competence, cohesion, low conflict and expression showed mixed findings. Specifically, the final model for *competence* (AIC = 448.03; intercept variance = .20, *P* < .01) showed a significant linear trend (Supplementary Table S7). Follow-up analyses showed baseline and 6-week (beta = .05, *P* = .30) and 6-week and 3-month end point (beta = .08, *P* = .07) scores were similar; however, baseline scores were significantly lower compared to the 3-month primary end point scores (beta = .08, *P* < .01). There were no condition effects. The model for *Cohesion* (AIC = 773.58; intercept variance = .23, *P* < .01) showed the linear trend was stable over time (Supplementary Table S8) and follow-up analyses showed that there were no condition effects. The model for *Low Conflict* (AIC = 681.49; intercept variance = .26, *P* < .01) showed a significant linear trend (Supplementary Table S9). Follow-up analyses showed baseline scores were similar to 6-weeks (beta = −.01, *P* = .89); however, 3-month primary end-point scores were significantly higher compared to baseline (beta = .06, *P* = .01) and 6-weeks (beta = .12, *P* = .02). There were no condition effects. *Expression* (AIC = 660.79; intercept variance = .26, *P* = .01) was stable over time (Supplementary Table S10). Follow-up analyses showed that participants in the team sport condition had significantly higher scores compared to the date night condition at baseline (beta = .21, *P* = .04; *d* = .34), 6-weeks (beta = .22, *P* = .03; *d* = .38) and was borderline at 3-month primary end-point (beta = .22, *P* = .06; *d* = .31).

### End of trial fidelity evaluation

Results of the end-of-trial fidelity evaluation can be found in Supplementary Table S11. Adherence to all three conditions was very good, with means in the “4” scoring, which equates to missing 1-2 sessions across the three months. There was no significant difference among groups. In terms of satisfaction with the activities, both the team sport condition and the “date night” control condition reported higher enjoyment and looking forward to the event each week more than the individual physical activity condition (*P* < .05); and mean scores were above “4,” indicating strong advocacy. All groups reported similar levels of benefit from participating in their respective conditions, with mean scores also above “4.” All groups also reported similar levels of challenges to engaging in the activities of their respective condition, yet the responses had mean scores in the “2” to “3” range, suggesting overall limited barriers to participation. Finally, most (77 to 79%) participants reported the intention to continue with the activities of their group condition now that the study had ended.

## Discussion

The purpose of this study was to evaluate the effectiveness of team sport participation on mental health (primary outcome) as well as other key psychosocial secondary outcomes (parenting stress, satisfaction with life, relationship satisfaction, family functioning) compared to an individual physical activity condition and a “date night” control condition among parents with children residing in the home. To our knowledge, this is the first experimental investigation into the effects of team sport participation on key psychosocial outcomes in any adult population. Average adherence to all three conditions was good (1-2 sessions missed across the three months; no significant difference between groups). Furthermore, the study featured viable and suitable comparator conditions (ie, “date night” or individual physical activity time), as they both represent other means of taking some personal time away from children to replenish and/or revitalize mental health and well-being. Thus, to our knowledge, this study represents the strongest test of the effects of adult team sport and physical activity participation on indicators of well-being in the contemporary scientific literature.

We hypothesized that the team sport condition would have better mental health and psychosocial outcomes at 3-months. This hypothesis was partially supported. Although the magnitude of change in mental health was nonsignificant over time for all 3 conditions, examination of the marginal means showed that the linear trend was increasing over time for the team sport condition, whereas the linear trend was declining for the date night and individual physical activity conditions. This change in different directions produced a significant and small-medium sized^66^ effect at 3-months such that the team sport participation condition had higher mental health than both the date-night comparison condition and the individual physical activity condition. There was no difference in mental health between the individual physical activity and date-night comparison groups. Our results provide some support for prior observational evidence for the link between sport participation and mental health^2,4,5,33^ and also some support for prior observational research that has identified team sport participation as more effective in promoting adult mental health compared to individual physical activity.^6^ These findings may be particularly important when considering parents of dependent children, who often experience challenges to maintaining their well-being during this stage in life.^19–22,67^ From a theoretical standpoint, it has been surmised that mental health benefits from sport participation are derived from the social nature of team sport.^2,68^ Our mental health measure in this study was the SF-12,^49^ which includes social functioning as a core composite, so our results lend some support for this theorizing.

Relatedly, we also found some support for our hypotheses that team sport participation would result in significant differences compared to the “date night” control condition and the individual physical activity condition on our secondary outcomes. Notably, parenting stress and relationship satisfaction (for those who reported being in a relationship) showed positive changes across time (parenting stress decreased across 3-months; relationship satisfaction increased from baseline to 6-weeks and then remained at the higher value 3-months later) for all groups. The team sport participation condition, however, showed more favorable small sized effects of lower parental stress compared to the “date night” control condition and higher effects on relationship satisfaction than the other two conditions. The family function variable of family expression (involving warmth, caring, and closeness) also showed specific small effect-sized benefits from the team sport participation condition in comparison to the “date night” control condition. Of note, these results did not support prior observational research suggesting that sport participation may lower stress compared to other forms of physical activity^7^; instead, our results indicate that the social components (social functioning in SF-12, relationship satisfaction) were better able to show benefits favoring team sport over individual physical activity.

Taken together, however, all of these conditions may be viable interventions to assist with alleviating parental distress and boosting relationship satisfaction, which have considerable challenges during the parenting years.^13,14,69–71^ Our end of trial data also showed that participants in all conditions reported strong advocacy in looking forward to the event each week, enjoyment of the activity and intention to continue, albeit that participants in the team sport and “date night” conditions reported higher enjoyment and to looking forward to the event each week more than those in the individual physical activity condition. As continued individual physical activity participation is often challenging for parents,^26–28,72,73^ the results of such advocacy for sport participation is encouraging. Sport participation may thus be a particularly valuable activity to recommend for this population based on these improved stress and social well-being outcomes and because the activity is so well received in terms of weekly enjoyment and expectations.

In contrast to the findings noted above, we showed that more generalized secondary psychosocial outcomes of overall satisfaction with life and family function had mixed findings, generally not supportive of our hypotheses. For example, overall satisfaction with life, parent perceptions of family health/competence, and low family conflict did improve significantly across the study, but there were no condition effects. It may be that because these conditions all feature a means of taking some personal time away from children to replenish and/or revitalize well-being, they may similarly impact perceptions of family health and life satisfaction. Overall, our results support the involvement of parents in any of these interventions for improving family health and generalized satisfaction with life.

Finally, perceptions of family cohesion was the only secondary outcome where we found no change or condition effect. In hindsight, this is unsurprising because cohesion is an assessment of coordinated parent and child activities.^55^ All of our conditions involved taking parents away from their children for a brief time each week, so it stands to reason why this family function measure was not impacted. By contrast, family-based physical activity interventions that involve parents and children being active *together*, show strong effects on cohesion.^74^ Thus, we recommend family-based interventions [see^75,76^] including all family members if the primary aim is to improve family function via cohesion.

Despite the considerable strengths of this study, there are noteworthy limitations that warrant mention. First, our end-of-trial data may be positively biased by those attendees who endured through the study compared to those who dropped out. Indeed, the team sport condition reported more drop out at 6-weeks than the other conditions and “scheduling issues” was a unique barrier to this condition, suggesting that the time confines of participating in team sport may not be fully accounted for in the end-of-trial assessment. Second, while a strength of our study was its pragmatism in accepting parents of all family systems and situations (ie, directly relevant to what a recreation program is likely to encounter with its registrants), the partially clustered data (some participants had partners, others did not) posed an analytical challenge. Our results used a dummy coded dyad variable as a covariate to “partially” capture this variance, yet we recommend a future study designed specifically to explore different parent family structures on these outcomes to better understand these potential effects. Third, our study was designed as our primary outcome measure (SF-12) and our collection of secondary outcome measures provide an array of personal and social well-being indices to explore the results of our study; however, future experimental research may benefit from specific measures of different forms of well-being (eg, by using SF 36) for additional accuracy. Fourth, our measures of fidelity to the different conditions were self-reported at our assessment end point, which may bias our understanding of how well participants actually adhered to their respective conditions. Our definitions of individual physical activity and team sport were also broad to account for the pragmatic design and the practicalities of accommodating individual and social physical activities in a community setting. We acknowledge this created some overlap in the boundaries of the conditions in a minority of cases. Future research may improve on this assessment by collecting adherence to condition data throughout the intervention and with direct markers (eg, program attendance, receipts from date night, etc.) and by narrowing the activities in each condition. Finally, our sample was moderately active (and thus likely possessed some motivation and prior sport experience), mainly white, middle income, and university educated. While many of these features do represent Greater Victoria,^77^ the generalizability of these results to more diverse socioeconomic conditions of parents is unknown. For example, parents of young children would likely have to procure childcare to participate in any of the conditions of this study, which may not be feasible for lower income parents, and differences in team sport motivation, experience, and/or ability, as well as opportunities to find a partner or team with which to co-participate, may also affect generalizability.

In summary, team sport participation over three months showed higher mental health (primary outcome) and relationship satisfaction compared to an individual physical activity condition and a “date night” control condition among parents with young children. Team sports participation also showed lower parental stress and family emotional expressiveness compared to the “date night” control condition. Taking part in all three conditions improved satisfaction with life, lowered stress, increased relationship satisfaction, benefited family system health/competence and lowered family conflict over time, presumably because all interventions allowed parents to spend some personal time away from children to revitalize. The findings, in particular, extend prior observational research on adult team sport participation by demonstrating this mode of activity may be useful to improve personal and social well-being. Team sport participation may be a viable activity to recommend for parents of children in the home, who are typically challenged by lower well-being, stress, and social isolation.

## Supplementary Material

kaae081_suppl_Supplementary_Tables

## Data Availability

De-identified data from this study are not available in an a public archive. De-identified data from this study will be made available (as allowable according to institutional IRB standards) by emailing the corresponding author.

## Transparency statements

For each of the five transparency practices, you will provide a statement that accurately describes your article. The following examples may be adapted for use. If none of the statements accurately describe your article, please provide a statement. **Statements should be provided on the cover page and will be published with your manuscript.**

1. *Study registration*. The clinical trial was pre-registered with the National Library of Medicine at the National Institutes of Health registration ID is NCT02898285, September of 2016.

2. *Analytic plan pre-registration*. The analysis plan was pre-registered with the National Library of Medicine at the National Institutes of Health registration ID is NCT02898285, September of 2016 and it is also included at: Grant, S., Beauchamp, M. R., Blanchard, C. M., Carson, V., & Rhodes, R. E. (2020). Promoting sport participation during early parenthood: A randomized controlled trial protocol. *Trials*, *21*, 1-10.

3. *Analytic code availability*. There is no analytic code associated with this study.

4. *Materials availability*. Materials used to conduct the study are presented at: Grant, S., Beauchamp, M. R., Blanchard, C. M., Carson, V., & Rhodes, R. E. (2020). Promoting sport participation during early parenthood: A randomized controlled trial protocol. *Trials*, *21*, 1-10.
