# Supplementary material for: Effect of recreational sport and physical activity participation on well-being during early parenthood: a randomized controlled trial
Source: Ann Behav Med. 2024 Dec 9;59(1):kaae081. doi: 10.1093/abm/kaae081 (PMC11783310; doi:10.1093/abm/kaae081)
Supplement: kaae081_suppl_Supplementary_Tables [file kaae081_suppl_supplementary_tables.docx]

Supplementary Table 1

Breakdown of Physical Activities Chosen by Participants from The Individual Physical Activity Group and The Sport Participation Group

| **Individual**  **Physical Activity Group Activities:** | **N** | **Sport**  **Participation Group Activities:** | **N** |
| --- | --- | --- | --- |
| **Drop In:** |  | **Registered Programs:** |  |
| Swimming | 13 | Badminton | 1 |
| Weight training | 8 | Ice Hockey | 2 |
| Yoga classes | 10 | Ball Hockey | 2 |
| Spin classes | 1 | Volleyball | 2 |
| Boxing | 1 | Curling | 2 |
| Climbing Gym | 1 | Masters Swim | 3 |
| Skating | 1 |  |  |
| **Other Activities Included:** |  | Bootcamp | 8 |
| Biking | 2 | Circuit Training | 5 |
| Walking | 4 | Dance Classes | 2 |
| Hiking | 3 | VSSC Multi Sports Teams | 4 |
| Running | 4 | Kick Boxing | 2 |
|  |  | Synchro Swimming | 2 |
|  |  | Running Clinic | 2 |
|  |  | Karate | 1 |
|  |  | Soccer | 2 |
|  |  | Bowling | 1 |

Supplementary Table 2

Marginal means and standard errors for the outcome variables by condition and time

|  | Control  Condition | Individual  Physical Activity | Team Sport  Participation |
| --- | --- | --- | --- |
| Variable | Mean (SE) | Mean (SE) | Mean (SE) |
| Mental Health |  |  |  |
| Baseline | 42.57 (1.85) | 44.59 (1.60) | 46.54 (1.77) |
| 6 weeks | 42.16 (1.22) | 44.02 (1.05) | 46.84 (1.12) |
| 3 months | 41.76 (1.28) | 43.45 (1.22) | 47.14 (1.37) |
| Satisfaction with Life^1^ |  |  |  |
| Baseline | 24.85 (1.37) | 27.28 (1.31) | 25.51 (1.36) |
| 6 weeks | 27.16 (1.30) | 28.83 (1.24) | 28.31 (1.28) |
| 3 months | 29.47 (1.46) | 30.38 (1.36) | 31.12 (1.56) |
| Parental Stress |  |  |  |
| Baseline | 2.44 (.06) | 2.43 (.05) | 2.29 (.05) |
| 6 weeks | 2.36 (.05) | 2.34 (.05) | 2.21 (.05) |
| 3 months | 2.28 (.06) | 2.24 (.05) | 2.12 (.06) |
| Relationship Satisfaction^1^ |  |  |  |
| Baseline | 27.63 (1.52) | 28.90 (1.37) | 32.94 (1.39) |
| 6 weeks | 29.14 (1.35) | 29.73 (1.27) | 33.92 (1.27) |
| 3 months | 30.65 (1.43) | 30.56 (1.52) | 34.90 (1.46) |
| Family Function: Competence^2^ |  |  |  |
| Baseline | 3.87 (.08) | 4.02 (.05) | 4.02 (.06) |
| 6 weeks | 3.95 (.07) | 4.07 (.05) | 4.08 (.05) |
| 3 months | 4.02 (.06) | 4.11 (.06) | 4.14 (.06) |
| Family Function: Cohesion |  |  |  |
| Baseline | 3.64 (.08) | 3.64 (.06) | 3.74 (.07) |
| 6 weeks | 3.67 (.08) | 3.69 (.05) | 3.78 (.07) |
| 3 months | 3.71 (.08) | 3.75 (.06) | 3.82 (.08) |
| Family Function: Low Conflict |  |  |  |
| Baseline | 4.02 (.08) | 4.14 (.07) | 4.06 (.08) |
| 6 weeks | 4.08 (.07) | 4.20 (.07) | 4.14 (.06) |
| 3 months | 4.14 (.07) | 4.27 (.07) | 4.21 (.07) |
| Family Function: Expression^2^ |  |  |  |
| Baseline | 4.16 (.08) | 4.34 (.06) | 4.37 (.07) |
| 6 weeks | 4.19 (.07) | 4.35 (.06) | 4.41 (.07) |
| 3 months | 4.23 (.08) | 4.37 (.07) | 4.45 (.09) |

Note. SE = standard error.

^1^ Marginal means are based on a squared distribution

^2^ Marginal means are based on a winsorized distribution

Supplementary Table 3

Effects sizes for condition comparisons based on the marginal means and standard deviations for a given outcome over time

|  | Baseline | 6 weeks | 3 months |
| --- | --- | --- | --- |
| Variable | *d* | *d* | *d* |
| Mental Health |  |  |  |
| Date Night vs. Individual PA | -.14 | -.20 | -.16 |
| Date Night vs. Team Sport | -.26 | -.48 | -.49 |
| Individual PA vs. Team Sport | -.14 | -.32 | -.35 |
| Satisfaction with Life^1^ |  |  |  |
| Date Night vs. Individual PA | -.22 | -.16 | -.08 |
| Date Night vs. Team Sport | -.06 | -.11 | -.13 |
| Individual PA vs. Team Sport | .16 | -.05 | -.06 |
| Parental Stress |  |  |  |
| Date Night vs. Individual PA | .02 | .05 | .09 |
| Date Night vs. Team Sport | .33 | .36 | .32 |
| Individual PA vs. Team Sport | .35 | .32 | .27 |
| Relationship Satisfaction^1^ |  |  |  |
| Date Night vs. Individual PA | -.11 | -.05 | .01 |
| Date Night vs. Team Sport | -.44 | -.44 | -.35 |
| Individual PA vs. Team Sport | -.36 | -.41 | -.36 |
| Family Function: Competence^2^ |  |  |  |
| Date Night vs. Individual PA | -.27 | -.23 | -.18 |
| Date Night vs. Team Sport | -.25 | -.25 | -.24 |
| Individual PA vs. Team Sport | .00 | -.02 | -.06 |
| Family Function: Cohesion |  |  |  |
| Date Night vs. Individual PA | .00 | -.04 | -.07 |
| Date Night vs. Team Sport | -.16 | -.18 | -.17 |
| Individual PA vs. Team Sport | -.19 | -.18 | -.12 |
| Family Function: Low Conflict |  |  |  |
| Date Night vs. Individual PA | -.19 | -.21 | -.22 |
| Date Night vs. Team Sport | -.06 | -.11 | -.12 |
| Individual PA vs. Team Sport | .13 | .01 | .11 |
| Family Function: Expression^2^ |  |  |  |
| Date Night vs. Individual PA | -.30 | -.29 | -.22 |
| Date Night vs. Team Sport | -.34 | -.38 | -.31 |
| Individual PA vs. Team Sport | -.06 | -.11 | -.12 |

Note. *d*  = ($\bar{X}$_1_ - $\bar{X}$_2_) / SD_pooled_

^1^ Effect size calculation based on a squared distribution

^2^ Effec size calculation based on a winsorized distribution

PA = Physical activity.

Supplementary Table 4

Results from the generalized linear mixed models for satisfaction with life

|  | Centered at Baseline | | |  | Centered at 6 weeks | | |  | Centered at 3 months | | |
| --- | --- | --- | --- | --- | --- | --- | --- | --- | --- | --- | --- |
| Parameter | Beta^1^ | 95% CI | |  | Beta^1^ | 95% CI | |  | Beta^1^ | 95% CI | |
| Intercept | 23.67** | 20.51, 26.82 | |  | 25.98** | 22.89, 29.05 | |  | 28.29** | 24.90, 31.67 | |
| Condition |  |  |  |  |  |  |  |  |  |  |  |
| Date Night | 0 |  |  |  | 0 |  |  |  | 0 |  |  |
| Individual PA | 2.43 | -1.31, 6.17 | |  | 1.67 | -1.88, 5.21 | |  | .91 | -3.03, 4.85 | |
| Team Sport^2,3,4^ | .66 | -3.13, 4.44 | |  | 1.15 | -2.42, 4.73 | |  | 1.65 | -2.53, 5.83 | |
| Linear Trend | 2.31** | 1.21, 3.41 | |  | 2.31 | 1.21, 3.41 | |  | -2.31** | -3.41, -1.21 | |
| Condition x Linear Trend |  |  |  |  |  |  |  |  |  |  |  |
| Date Night x Linear Trend | 0 |  |  |  | 0 |  |  |  | 0 |  |  |
| Individual PA x Linear Trend | -.76 | -2.24, .71 | |  | -.76 | -2.24, .71 | |  | .76 | -.71, 2.24 | |
| Team Sport x Linear Trend | .49 | -1.28, 2.26 | |  | .49 | -1.28, 2.26 | |  | -.49 | -2.26, 1.28 | |
| Dyad |  |  |  |  |  |  |  |  |  |  |  |
| Do not have a partner in study | 0 |  |  |  | 0 |  |  |  | 0 |  |  |
| Have a partner in study | 2.37 | -.54, 5.28 | |  | 2.37 | -.54, 5.28 | |  | 2.37 | -.54, 5.28 | |

**Note.** *p < .05; **p < .01; CI = confidence interval. ^1^ Betas are based on a squared distribution. PA = Physical activity.

**Pairwise Comparisons by Condition**

^2^ At baseline, Team Sport = Individual PA (contrast estimate = -1.77, 95% CI [-5.50, 1.96]).

^3^ At 6 weeks, Team Sport = Individual PA (contrast estimate = -.51, 95% CI [-4.05, 3.02]).

^4^ At 3 months, Team Sport = Individual PA (contrast estimate = .74, 95% CI [-3.34, 4.83]).

Supplemental Table 5

Results from generalized linear mixed models for perceived stress

|  | Centered at Baseline | | |  | Centered at 6 weeks | | |  | Centered at 3 months | | |
| --- | --- | --- | --- | --- | --- | --- | --- | --- | --- | --- | --- |
| Parameter | Beta^1^ | 95% CI | |  | Beta^1^ | 95% CI | |  | Beta^1^ | 95% CI | |
| Intercept | 2.51** | 2.38, 2.65 | |  | 2.43** | 2.31, 2.56 | |  | 2.36** | 2.23, 2.48 | |
| Condition |  |  |  |  |  |  |  |  |  |  |  |
| Date Night | 0 |  |  |  | 0 |  |  |  | 0 |  |  |
| Individual PA | -.01 | -.17, .15 | |  | -.03 | -.17, .11 | |  | -.04 | -.19, .10 | |
| Team Sport^2,3,4^ | -.15 | -.31, .02 | |  | -.16* | -.30, -.01 | |  | -.17* | -.33, -.004 | |
| Linear Trend | -.08 | -.13, -.03 | |  | -.08 | -.13, -.03 | |  | .08 | .03, .13 | |
| Condition x Linear Trend |  |  |  |  |  |  |  |  |  |  |  |
| Date Night x Linear Trend | 0 |  |  |  |  |  |  |  | 0 |  |  |
| Individual PA x Linear Trend | -.02 | -.08, .05 | |  | -.02 | -.08, .05 | |  | .02 | -.05, .08 | |
| Team Sport x Linear Trend | -.01 | -.08, .06 | |  | -.01 | -.08, .06 | |  | .01 | -.06, .08 | |
| Dyad |  |  |  |  |  |  |  |  |  |  |  |
| Do not have a partner in study | 0 |  |  |  |  |  |  |  | 0 |  |  |
| Have a partner in study | -.14* | -.26, -.03 | |  | -.14* | -.26, -.03 | |  | -.14* | -.26, -.03 | |

**Note.** *p < .05; **p < .01; CI = confidence interval. ^1^ Estimates are based on the original distribution. PA = Physical activity.

**Pairwise Comparisons by Condition**

^2^ At baseline, Team Sport = Individual PA (contrast estimate = -.14, 95% CI [-.28, .01]).

^3^ At 6 weeks, Team Sport = Individual PA (contrast estimate = -.13, 95% CI [-.27, .01]).

^4^ At 3 months, Team Sport = Individual PA (contrast estimate = -.12, 95% CI [-.28, .03]).

Supplemental Table 6

Results from generalized linear mixed models for relationship satisfaction

|  | Centered at Baseline | | |  | Centered at 6 weeks | | |  | Centered at 3 months | | |
| --- | --- | --- | --- | --- | --- | --- | --- | --- | --- | --- | --- |
| Parameter | Beta^1^ | 95% CI | |  | Beta^1^ | 95% CI | |  | Beta^1^ | 95% CI | |
| Intercept | 25.92** | 22.41, 29.43 | |  | 27.43** | 24.19, 30.67 | |  | 28.94** | 25.59, 32.30 | |
| Condition |  |  |  |  |  |  |  |  |  |  |  |
| Date Night | 0 |  |  |  | 0 |  |  |  | 0 |  |  |
| Individual PA | 1.27 | -2.75, 5.29 | |  | .59 | -3.07, 4.25 | |  | -.09 | -4.19, 4.01 | |
| Team Sport^2,3,4^ | 5.31* | 1.29, 9.32 | |  | 4.78* | 1.15, 8.41 | |  | 4.25* | .26, 8.25 | |
| Linear Trend | 1.51* | .37, 2.66 | |  | 1.51 | .37, 2.66 | |  | -1.51 | -2.66, -.37 | |
| Condition x Linear Trend |  |  |  |  |  |  |  |  |  |  |  |
| Date Night x Linear Trend | 0 |  |  |  |  |  |  |  | 0 |  |  |
| Individual PA x Linear Trend | -.68 | -2.45, 1.09 | |  | -.68 | -2.45, 1.09 | |  | .68 | -1.09, 2.45 | |
| Team Sport x Linear Trend | -.53 | -2.23, 1.18 | |  | -.53 | -2.23, 1.18 | |  | .53 | -1.18, 2.23 | |
| Dyad |  |  |  |  |  |  |  |  |  |  |  |
| Do not have a partner in study | 0 |  |  |  |  |  |  |  | 0 |  |  |
| Have a partner in study | 3.41* | .41, 6.42 | |  | 3.41* | .41, 6.42 | |  | 3.41* | .41, 6.42 | |

**Note.** *p < .05; **p < .01; CI = confidence interval. ^1^ Estimates are based on a squared distribution. PA = Physical activity.

**Pairwise Comparisons by Condition**

^2^ At baseline, Team Sport > Individual PA (contrast estimate = 4.03*, 95% CI [.18, 7.88]).

^3^ At 6 weeks, Team Sport > Individual PA (contrast estimate = 4.19*, 95% CI [.62, 7.78]).

^4^ At 3 months, Team Sport > Individual PA (contrast estimate = 4.34*, 95% CI [.18, 8.50]).

Supplemental Table 7

Results from generalized linear mixed models for perceived competence

|  | Centered at Baseline | | |  | Centered at 6 weeks | | |  | Centered at 3 months | | |
| --- | --- | --- | --- | --- | --- | --- | --- | --- | --- | --- | --- |
| Parameter | Beta^1^ | 95% CI | |  | Beta^1^ | 95% CI | |  | Beta^1^ | 95% CI | |
| Intercept | 3.81** | 3.64, 3.97 | |  | 3.88** | 3.74, 4.03 | |  | 3.96** | 3.82, 4.10 | |
| Condition |  |  |  |  |  |  |  |  |  |  |  |
| Date Night | 0 |  |  |  | 0 |  |  |  | 0 |  |  |
| Individual PA | .15 | -.03, .34 | |  | .12 | -.04, .28 | |  | .09 | -.08, .25 | |
| Team Sport^2,3,4^ | .15 | -.04, .34 | |  | .13 | -.03, .30 | |  | .12 | -.05, .28 | |
| Linear Trend | .08** | .03, .12 | |  | .08** | .03, .12 | |  | -.08** | -.12, -.03 | |
| Condition x Linear Trend |  |  |  |  |  |  |  |  |  |  |  |
| Date Night x Linear Trend | 0 |  |  |  |  |  |  |  | 0 |  |  |
| Individual PA x Linear Trend | -.03 | -.10, .03 | |  | -.03 | -.10, .03 | |  | .03 | -.03, .10 | |
| Team Sport x Linear Trend | -.02 | -.09, .05 | |  | -.02 | -.09, .05 | |  | .02 | -.05, .09 | |
| Dyad |  |  |  |  |  |  |  |  |  |  |  |
| Do not have a partner in study | 0 |  |  |  |  |  |  |  | 0 |  |  |
| Have a partner in study | .13 | -.01, .26 | |  | .13 | -.01, .26 | |  | .13 | -.01, .26 | |

**Note.** *p < .05; **p < .01; CI = confidence interval. ^1^ Estimates are based on a winsorized distribution. PA = Physical activity.

**Pairwise Comparisons by Condition**

^2^ At baseline, Team Sport = Individual PA (contrast estimate = -.003, 95% CI [-.16, .15]).

^3^ At 6 weeks, Team Sport = Individual PA (contrast estimate = .01, 95% CI [-.13, .16]).

^4^ At 3 months, Team Sport = Individual PA (contrast estimate = .03, 95% CI [-.13, .19]).

Supplemental Table 8

Results from generalized linear mixed models for perceived cohesion

|  | Centered at Baseline | | |  | Centered at 6 weeks | | |  | Centered at 3 months | | |
| --- | --- | --- | --- | --- | --- | --- | --- | --- | --- | --- | --- |
| Parameter | Beta^1^ | 95% CI | |  | Beta^1^ | 95% CI | |  | Beta^1^ | 95% CI | |
| Intercept | 3.54** | 3.37, 3.72 | |  | 3.57** | 3.41, 3.73 | |  | 3.61** | 3.44, 3.78 | |
| Condition |  |  |  |  |  |  |  |  |  |  |  |
| Date Night | 0 |  |  |  | 0 |  |  |  | 0 |  |  |
| Individual PA | .001 | -.20, .21 | |  | .02 | -.16, .20 | |  | .04 | -.16, .24 | |
| Team Sport^2,3,4^ | .10 | -.13, .32 | |  | .10 | -.09, .30 | |  | .11 | -.12, .34 | |
| Linear Trend | .03 | -.03, .10 | |  | .03 | -.03, .10 | |  | -.03 | -.10, .03 | |
| Condition x Linear Trend |  |  |  |  |  |  |  |  |  |  |  |
| Date Night x Linear Trend | 0 |  |  |  | 0 |  |  |  | 0 |  |  |
| Individual PA x Linear Trend | .02 | -.07, .11 | |  | .02 | -.07, .11 | |  | -.02 | -.11, .07 | |
| Team Sport x Linear Trend | .01 | -.10, .12 | |  | .01 | -.10, .12 | |  | -.01 | -.12, .10 | |
| Dyad |  |  |  |  |  |  |  |  |  |  |  |
| Do not have a partner in study | 0 |  |  |  | 0 | 0 | |  | 0 |  |  |
| Have a partner in study | .20* | .05, .35 | |  | .20 | .05, .35 | |  | .20 | .05, .35 | |

**Note.** *p < .05; **p < .01; CI = confidence interval. ^1^ Estimates are based on the original distribution. PA = Physical activity.

**Pairwise Comparisons by Condition**

^2^ At baseline, Team Sport = Individual PA (contrast estimate = .09, 95% CI [-.09, .28]).

^3^ At 6 weeks, Team Sport = Individual PA (contrast estimate = .08, 95% CI [-.08, .25]).

^4^ At 3 months, Team Sport = Individual PA (contrast estimate = .07, 95% CI [-.13, .28]).

Supplemental Table 9

Results from generalized linear mixed models for perceived conflict

|  | Centered at Baseline | | |  | Centered at 6 weeks | | |  | Centered at 3 months | | |
| --- | --- | --- | --- | --- | --- | --- | --- | --- | --- | --- | --- |
| Parameter | Beta^1^ | 95% CI | |  | Beta^1^ | 95% CI | |  | Beta^1^ | 95% CI | |
| Intercept | 3.98** | 3.80, 4.16 | |  | 4.04** | 3.88, 4.21 | |  | 4.10** | 3.94, 4.27 | |
| Condition |  |  |  |  |  |  |  |  |  |  |  |
| Date Night | 0 |  |  |  | 0 |  |  |  | 0 |  |  |
| Individual PA | .12 | -.09, .33 | |  | .12 | -.06, .31 | |  | .13 | -.06, .32 | |
| Team Sport^2,3,4^ | .04 | -.17, .26 | |  | .06 | -.13, .24 | |  | .07 | -.12, .27 | |
| Linear Trend | .06* | .02, .11 | |  | .06* | .02, .11 | |  | -.06* | -.11, .02 | |
| Condition x Linear Trend |  |  |  |  |  |  |  |  |  |  |  |
| Date Night x Linear Trend | 0 |  |  |  | 0 |  |  |  | 0 |  |  |
| Individual PA x Linear Trend | .004 | -.07, .07 | |  | .004 | -.07, .07 | |  | -.004 | -.07, .07 | |
| Team Sport x Linear Trend | .02 | -.07, .11 | |  | .02 | -.07, .11 | |  | -.02 | -.11, .07 | |
| Dyad |  |  |  |  |  |  |  |  |  |  |  |
| Do not have a partner in study | 0 |  |  |  | 0 |  | |  | 0 |  |  |
| Have a partner in study | .07 | -.09, .23 | |  | .07 | -.09, .23 | |  | .07 | -.09, .23 | |

**Note.** *p < .05; **p < .01; CI = confidence interval. ^1^ Estimates are based on the original distribution. PA = Physical activity.

**Pairwise Comparisons by Condition**

^2^ At baseline, Team Sport = Individual PA (contrast estimate = -.08, 95% CI [-.29, .13]).

^3^ At 6 weeks, Team Sport = Individual PA (contrast estimate = -.07, 95% CI [-.25, .11]).

^4^ At 3 months, Team Sport = Individual PA (contrast estimate = -.05, 95% CI [-.25, .14]).

Supplemental Table 10

Results from generalized linear mixed models for perceived expression

|  | Centered at Baseline | | |  | Centered at 6 weeks | | |  | Centered at 3 months | | |
| --- | --- | --- | --- | --- | --- | --- | --- | --- | --- | --- | --- |
| Parameter | Beta^1^ | 95% CI | |  | Beta^1^ | 95% CI | |  | Beta^1^ | 95% CI | |
| Intercept | 4.10** | 3.92, 4.27 | |  | 4.13** | 3.96, 4.29 | |  | 4.16** | 3.98, 4.34 | |
| Condition |  |  |  |  |  |  |  |  |  |  |  |
| Date Night | 0 |  |  |  | 0 |  |  |  | 0 |  |  |
| Individual PA | .18 | -.02, .37 | |  | .16 | -.02, .34 | |  | .14 | -.06, .34 | |
| Team Sport^2,3,4^ | .21* | .01, .41 | |  | .22* | .02, .41 | |  | .22 | -.01, .46 | |
| Linear Trend | .03 | -.03, .09 | |  | .03 | -.03, .09 | |  | -.03 | -.09, .03 | |
| Condition x Linear Trend |  |  |  |  |  |  |  |  |  |  |  |
| Date Night x Linear Trend | 0 |  |  |  | 0 |  |  |  | 0 |  |  |
| Individual PA x Linear Trend | -.02 | -.09, .06 | |  | -.02 | -.09, .06 | |  | .02 | -.06, .09 | |
| Team Sport x Linear Trend | .01 | -.09, .10 | |  | .01 | -.09, .10 | |  | -.01 | -.10, .09 | |
| Dyad |  |  |  |  |  |  |  |  |  |  |  |
| Do not have a partner in study | 0 |  |  |  | 0 |  | |  | 0 |  |  |
| Have a partner in study | .13 | -.02, .29 | |  | .13 | -.02, .29 | |  | .13 | -.02, .29 | |

**Note.** *p < .05; **p < .01; CI = confidence interval. ^1^ Estimates are based on a winsorized distribution.

**Pairwise Comparisons by Condition**

^2^ At baseline, Team Sport = Individual PA (contrast estimate = .03, 95% CI [-.14, .21]).

^3^ At 6 weeks, Team Sport = Individual PA (contrast estimate = .06, 95% CI [-.13, .24]).

^4^ At 3 months, Team Sport = Individual PA (contrast estimate = .08, 95% CI [-.15, .31]).

Supplemental Table 11

Descriptive statistics for end-of-trial evaluation among participants.

| Variable | Team Sport | Individual PA | Date Night | F_2,146_ | p | *η^2^* |
| --- | --- | --- | --- | --- | --- | --- |
|  | Mean ± SD | Mean ± SD | Mean ± SD |  |  |  |
| Adherence to the condition | 4.17 ± 0.86 | 3.87 ± 0.88 | 4.08 ± 0.97 | 1.28 | .28 | .02 |
| Enjoyment | 4.49 ± .0.64_a_ | 4.15 ± 0.81_a,b_ | 4.52 ± 0.77_b_ | 3.79 | .03 | .05 |
| Benefit | 4.44 ± 0.59 | 4.30 ± 0.78 | 4.48 ± 0.73 | 0.94 | .39 | .01 |
| Looked forward to each session | 4.37 ± 0.77 _a_ | 3.89 ± 0.98 _a,b_ | 4.36 ± 0.78 _b_ | 4.88 | .01 | .06 |
| Challenging to attend | 2.85 ± 1.44 | 3.15 ± 1.25 | 2.98 ± 1.28 | 0.56 | .57 | .01 |
|  |  |  |  |  |  |  |
| % Intention to continue | 78% | 77% | 79% | χ^2^ (2) = .09 | .96 |  |

Note. SD = standard deviation. Subscript matches represent significant differences (*p* < .05) using LSD post hoc tests. PA = Physical activity.
